# Rethinking desert definitions: Bridging the gap between science, policy, and conservation

**DOI:** 10.1007/s13280-025-02276-9

**Published:** 2025-11-07

**Authors:** Amir Lewin, Gopal Murali, Uri Roll, Troy Sternberg, Shimon Rachmilevitch

**Affiliations:** 1 French Associates Institute for Agriculture and Biotechnology of Drylands, Jacob Blaustein Institutes for Desert Research, Ben-Gurion University of the Negev, 849900 Midreshet Ben-Gurion, Israel; 2 Department of Ecology and Evolutionary Biology, University of Arizona, Tucson, AZ 85721 USA; 3 Mitrani Department of Desert Ecology, The Jacob Blaustein Institutes for Desert Research, Ben-Gurion University of the Negev, 8499000 Midreshet Ben-Gurion, Israel; 4 School of Geography, University of Oxford, Oxford, OX1 3QY UK; 5 Goldman Sonnenfeldt School of Sustainability and Climate Change, Ben-Gurion University of the Negev, 8410501 Beer-Sheva, Israel

**Keywords:** Desert classifications, Desertification, Drylands, Land degradation, Policy implications, Scientific communication

## Abstract

Deserts are often misperceived as desolate, non-productive landscapes. This perception contributes to the misuse of terms like ‘semiarid’ and ‘desertification’, creating ambiguity in how deserts are defined and geographically delineated. Such conceptual confusion can hinder effective policy by obscuring what constitutes a desert and where they occur. We identify key incongruencies between climatic and ecological approaches to classifying deserts, revealing nearly 12 million km^2^ of distinct, non-overlapping desert regions globally. A new, more comprehensive desert classification is needed to better support policy goals in desert regions. This refined understanding is especially urgent as human pressures accelerate land degradation across drylands—a process the United Nations Convention to Combat Desertification refers to as ‘desertification’. However, the term is misperceived as the expansion of deserts while ignoring land degradation in actual desert regions. We challenge these misconceptions and advocate for a more nuanced understanding of deserts to strengthen scientific and policy frameworks.

**Supplementary Information:**

The online version contains supplementary material available at 10.1007/s13280-025-02276-9.

## Introduction

Deserts are vital habitats supporting diverse landscapes and endemic species, as well as providing key ecosystem services (Maestre et al. 2021; Chen and Constanza 2024). People have inhabited deserts for millennia—environments that have had a profound influence on the development of human society (UNEP 2006). Deserts continue to play an important role in the world’s environmental and social heritage, encompassing unique biodiversity as well as notable urban, agricultural, energy, and cultural centers (Goudie 2002; Sagie et al. 2013; Zhang et al. 2023). Yet despite their significance, deserts are often perceived as desolate and non-productive landscapes. While deserts do have low primary productivity due to water scarcity and extreme conditions, this differs from cultural and policy narratives that portray them as lifeless or barren. In fact, deserts are geographically diverse, ecologically rich, and culturally significant regions that contribute to global diversity (Maestre et al. 2021). It is unfortunate that the world’s largest biome is synonymous with these and other negative connotations, such as ‘empty’ or ‘uninhabited.’ Consequently, deserts are overlooked in broad conservation frameworks and development priorities (Lewin et al. 2024). Furthermore, the precise geographic delineation of deserts is unclear due to different approaches in classification based on different criteria (for example, climatic versus ecological; UNEP 2006). In addition, the imprecise use of terms such as ‘semiarid’ and ‘drylands’ when referring to deserts contributes to confusion regarding the specific characteristics of these regions (for example, Bai et al. 2021), particularly in policy and management contexts. Moreover, deserts are increasingly framed as regions to transform rather than preserve, as evident by varied greening and afforestation initiatives that aim ‘to make the desert bloom’ (Sagie et el. 2013; Sternberg 2015; Turner et al. 2023). These programs often extend into true desert regions (Naia et al. 2021; Licata et al. 2025), reinforcing the misconception of natural deserts as degraded landscapes in need of repair. Deserts are especially sensitive to these and other increasing human land-use pressures through conversion for agriculture and alternative energy sources (that is, solar panels and bioenergy), mining and development, overgrazing, invasive species, and climate change—pressures that are expected to intensify in the coming decades (Lewin et al. 2024). Human pressures in deserts may result in severe land degradation, a process referred to as ‘desertification’—possibly one of the most important global environmental change issues, threatening desert ecosystems and human well-being (MEA 2005; Reynolds et al. 2007; Grainger et al. 2009; Prăvălie 2016; Burrell and De Kauwe 2020; Mirzabaev 2022; Zhang et al. 2023). However, the official UN definition of desertification focuses only on drylands while explicitly excluding vast areas of hyperarid deserts, overlooking degradation in these sensitive and diverse regions. Unfortunately, the term desertification is also misperceived as the physical spread of deserts or the conversion of non-desert biomes (Grainger 2009)—a semantic ambiguity, as the UN definition refers strictly to land degradation within drylands, not desert expansion. As a result, deserts are further infused with negative associations, hindering policy actions and effective development initiatives. Here, we review two commonly used approaches to classifying deserts to identify key incongruencies between desert delineations, while also highlighting the diverse and vital human social and ecological systems that non-overlapping desert regions support. We then evaluate the usage of the term desertification and its efficacy in managing degradation across desert regions inclusively. Recognizing these distinctions is crucial for accurate and effective land management strategies in desert systems.

### Comparing desert classifications

**Fig. 1:**
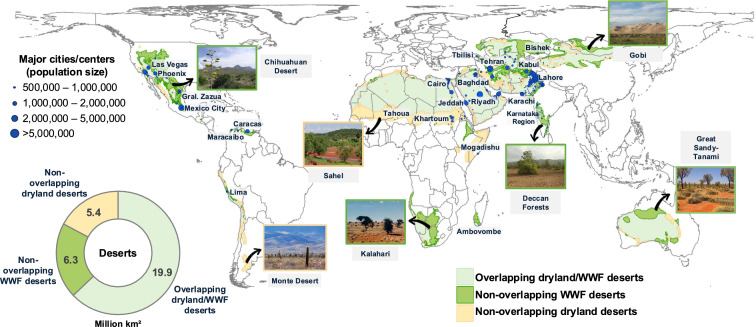
Comparing the geographic distribution of desert classifications: dryland deserts (‘hyperarid’ and ‘arid’ dryland subtypes; UNEP-WCMC) and WWF deserts (‘Deserts & Xeric Shrublands biome’; WWF). Dryland deserts are classified using a climatic approach based on aridity; WWF deserts are classified using an ecological approach. The donut shows the relative area of overlapping and non-overlapping desert regions (million km^2^). Light-green = overlapping dryland and WWF deserts; dark-green = non-overlapping WWF deserts; light-orange = non-overlapping dryland deserts. Photograph insets show distinct desert regions not considered by both the WWF and UNEP-WCMC. Deserts are generally categorized as hot, cold, coastal, sclerophyllous, and succulent desert types. Blue circles show major human population centers by administration units (> 500,000 people; data from Gridded Population of the World, Version 4; 2020). Image inset references and source attributions are listed below the references

Deserts are generally characterized as regions with extreme water deficits and low productive biological systems. However, deserts are classified according to several criteria (Vicente-Serrano et al. 2024). One method uses a climatic approach based on aridity, defined as the ratio between annual precipitation and potential evapotranspiration. For example, the UN Environment Programme–World Conservation Monitoring Centre (UNEP-WCMC) and the UN Convention to Combat Desertification (UNCCD) use this aridity index (AI) to classify ‘drylands’—areas with AI below 0.65 (UNEP-WCMC 2007). Here, deserts are defined as ‘hyperarid’ and ‘arid’ dryland subtypes (UNEP 2006)—areas with AI below 0.2—that is, where rainfall is less than 20% of evaporative demand (herein ‘dryland deserts’); hyperarid areas (AI < 0.05) are excluded from formal desertification assessments. A second measure for classifying deserts uses an ecological approach. For example, the World Wildlife Fund for Nature (WWF) classifies ‘Deserts and Xeric Shrublands’ according to biogeographic and biophysical features (Sorenson 2007; Dinerstein et al. 2017). This classification refers to land units containing a distinct desert assemblage of natural biotic communities with shared environmental conditions (herein ‘WWF deserts’). Comparing these different desert classifications allows us to highlight their complementarity and to identify unique desert regions within respective classifications (Sorenson 2007). A similar global comparison was conducted in the Global Deserts Outlook (UNEP 2006), which found general alignment between desert delineations. Our analysis builds on this, while quantifying the substantial geographic incongruencies (nearly 12 million km^2^) between climatic and ecological classifications, highlighting implications for conservation and policy (Fig. 1). The overlap of WWF deserts and dryland deserts sums to an area of about 19.9 million km^2^, representing where the two delineations intersect (Fig. 1). However, an additional area of 6.3 million km^2^ of WWF deserts lies outside of dryland deserts, and an additional area of 5.4 million km^2^ of dryland deserts does not overlap with WWF deserts. Therefore, climatic and ecological criteria for classifying deserts are not fully compatible geographically, revealing vast distinct and diverse desert regions not considered by both the WWF and UNEP-WCMC, encompassing hot, cold/inland, coastal, sclerophyllous, and succulent desert types and a range of climate zones. These non-overlapping deserts cover large areas of the Kalahari and Sahel regions in Africa, Deccan Forests and Gobi Desert in Asia, Chihuahuan Desert of North America, and Great Sandy-Tanami Desert of Australia (Fig. 1). Extensive geographic incongruencies between disparate desert delineations may have important implications for guiding management, conservation, and policy goals in deserts.

### Policy implications for human land-use and biodiversity conservation in deserts

Deserts support about 1 billion people and serve as vital urban, agricultural, energy, tourism, and scientific centers globally (Table 1). Large population clusters are prevalent in deserts across India and Pakistan, Iran, Saudi Arabia, and Egypt—including major cities located in non-overlapping deserts (for example, Mexico City in WWF deserts, and Khartoum in dryland deserts; Fig. 1). We evaluated human land-use differences between desert classifications. Asian deserts combined are home to 694 million people and more than 1 million km^2^ of agricultural and urban land areas (Table 1). Notably, a considerably larger proportion of WWF deserts in Asia have been converted for human land uses compared to overlapping desert regions and dryland deserts. For example, more than 22% of WWF deserts in Asia are used for crops (~ 611 661 km^2^), compared to only about 5% of overlapping deserts (~ 384 909 km^2^) and 7% of dryland deserts (~ 68 368 km^2^) in Asia. This may reflect the fact that many WWF deserts not overlapping with dryland deserts fall outside aridity thresholds defined by UNEP-WCMC and thus occur in relatively more humid, populated regions subject to greater human pressure. This suggests that ecologically defined deserts may require distinct management approaches that account for higher human presence and intensified land use, despite not being classified as climatically arid. In Africa and Asia, which contain most global deserts, human populations and development rates are projected to increase dramatically faster compared to other regions. Projections indicate that many desert regions within Africa and Asia are also expected to experience substantial increases in population and land-use pressure, driven by the expansion of agriculture, urban development, infrastructure, mining and energy projects (Seto et al. 2012; Mansour et al. 2022; Lewin et al. 2024). These localized pressures are likely to intensify existing vulnerabilities and accelerate land degradation in desert systems (Portnov and Hare 2012; Cherlet et al. 2018). Such pressures already present significant challenges in terms of climate migration, poverty, food/water security, and conflict (Barbier and Hochard 2018). Land-use pressures are also considerable in many developed desert regions (for example, Phoenix, Arizona) due to extensive agriculture, urban and other human land uses.

**Table 1:** Human land-use patterns and populations in WWF and dryland deserts by continent. Crops area (i.e., human planted cereals, grasses and crops) and built area (i.e., human structures, major road and rail networks, etc.). Parentheses show proportion of land cover (%) in respective overlapping and non-overlapping desert areas. Data from Sentinel-2 10m land-use/land-cover time series (2023). Human population data from Gridded Population of the World, Version 4 (2020)

	Crops area (km^2^)			Built area (km^2^)			Human populations (millions)		
	Overlapping deserts	Non-overlapping WWF deserts	Non-overlapping dryland deserts	Overlapping deserts	Non-overlapping WWF deserts	Non-overlapping dryland deserts	Overlapping deserts	Non-overlapping WWF deserts	Non-overlapping dryland deserts
Africa	36 795 (0.44)	31 255 (29)	98 636 (72)	6481 (0.08)	3657 (0.34)	18 114 (0.5)	30.0	18.9	147.9
Asia	384 909 (4.7)	611 661 (22.3)	68 368 (7.1)	82 363 (1.0)	74 952 (27)	15 397 (1.6)	255.9	388.9	49.3
Australia	815 (0.03)	1339 (0.18)	1206 (0.49)	318 (0.01)	122 (0.02)	44 (0.02)	0.11	0.076	0.002
North America	31 660 (4.5)	80 711 (5.4)	30 659 (19.5)	11 418 (1.6)	29 504 (2.0)	3218 (2.0)	18.1	58.6	5.4
South America	8028 (3.1)	3598 (2.3)	8773 (2.0)	3734 (1.5)	3993 (2.5)	4005 (0.93)	18.4	18.4	6.4
Europe	1283 (5.0)	1478 (2.4)	470 (7.1)	442 (1.7)	106 (0.17)	30 (0.46)	0.77	0.15	0.051
Global	462 480 (2.3)	730 042 (11.6)	208 112 (3.8)	104 756 (0.5)	112 334 (1.8)	40 808 (0.7)	323.2	485.0	208.9

Beyond classification, the resilience of different desert systems to diverse land-use pressures may be a meaningful basis for informing management (Turner et al. 2003, 2007; Abuzaid et al. 2021). Deserts vary widely in their ecological and socioeconomic contexts, which shape how they respond to stressors such as agriculture, infrastructure, and urban expansion. For instance, while overuse of agricultural inputs such as fertilizer and water resources drives degradation in some Asian deserts, underuse and lack of access to resources may constrain productivity in African deserts—each posing distinct, long-term challenges (IPBES 2018). Emerging global data and convergence-of-evidence approaches, as presented in the World Atlas of Desertification (WAD), now enable more spatially nuanced insights into these dynamics, underscoring the need for land-use strategies tailored to the specific vulnerabilities and adaptive capacities of different desert systems (Cherlet et al. 2018).

**Fig. 2:**
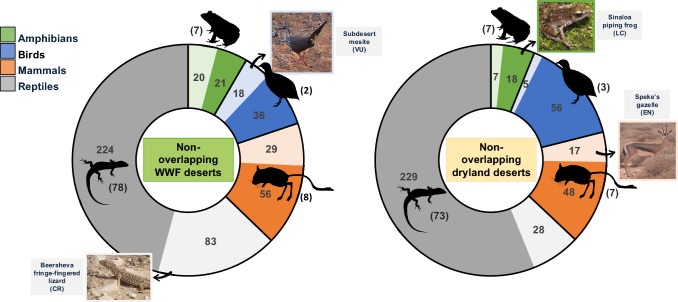
Amphibian, bird, mammal, and reptile species in non-overlapping WWF and dryland deserts (i.e., species ≥ 50% of global distribution range found in non-overlapping deserts only). Green = amphibians; blue = birds; orange = mammals; gray = reptiles. Respective light colors per taxon correspond to number of threatened species (i.e., IUCN categories ‘CR’, ‘EN’, ‘VU’). Parentheses = number of endemic species per taxon (that is, > 99% global range found in non-overlapping deserts only). Silhouette images of vertebrate taxa were obtained without changes from PhyloPic (https://www.phylopic.org/). Image inset references and source attributions are listed below the references

Deserts are also home to a rich variety of unique and endemic plant and animal species due to wide-ranging behavioural, morphological and physiological adaptations to extreme climatic conditions over heterogeneous landscapes and long evolutionary histories (Maestre et al. 2021; Gross et al. 2024). However, deserts are under-protected globally compared to other regions, with an even lower proportion of protected areas designated for biodiversity conservation (Lewin et al. 2024). For example, a total of about 487 vertebrate species have most of their global range (that is, 50% or more) in areas unique to WWF deserts not overlapping with dryland deserts (Fig. 2). Many of these species are classified as threatened by the International Union for Conservation of Nature (IUCN) and endemic to these regions alone. Similarly, about 408 vertebrate species are found mostly in non-overlapping dryland deserts. Non-overlapping deserts are certain to comprise a considerably higher diversity of endemic vascular and nonvascular plant species and soil microorganisms encompassing unique forms and functional traits (Maestre et al. 2021; Gross et al. 2024), which should be incorporated in large-scale desert conservation frameworks. Therefore, how we define and classify deserts is crucial for guiding land-use management and addressing the challenges of human development and environmental pressures. Ensuring deserts are ecologically well-represented is key to preserving biodiversity, promoting sustainable land use, and reaching global targets of protection (that is, 30% coverage of ecologically representative lands by 2030; CBD 2022).

### A new framework to classify deserts

It appears that a climatic approach based strictly on aridity to categorize deserts fails to fully capture the diversity and complexity of recognized desert regions (Figs. 1 and 2). Similarly, a strictly ecological approach categorizing deserts omits several distinct regions. UNEP’s reliance on rigid climatic thresholds, particularly aridity indices, imposes overly simplistic boundaries that fail to capture the complex ecological gradients and transitional zones that define many desert regions. This methodological limitation explains much of the discrepancy between UNEP’s and WWF’s desert classifications. Therefore, a range of climatic and biophysical attributes essential for classifying deserts should provisionally include among other variables: precipitation seasonality, climatic variability, rainfall pulses, growing period, functional diversity, and desert ecosystem mechanisms (for example, biological soil-crust formation) (Kottek et al. 2006; Sorenson 2007; FAO and IIASA 2021; Spinoni et al. 2021; Grunzweig et al. 2022). A more comprehensive desert classification might be developed by layering these variables onto existing delineations using remote sensing and GIS-based approaches, similar to how multifactorial definitions have been applied to classify other regions (Feeley and Stroud 2018). Non-desert drylands comprise ‘semiarid’ and ‘dry subhumid’ subtypes—areas with an aridity ratio (AI) of up to 0.65 (that is, a water deficit at least 1.5 times greater than precipitation). Dryland subtypes combined (that is, hyperarid, arid, semiarid and dry subhumid) encompass all of WWF deserts and about 42% of the earth’s terrestrial surface, and a third of the global human population. However, when referring to deserts it is crucial to distinguish between potentially over-inclusive ‘drylands’ and the overuse of the term ‘desert’ when referring to drylands. The lack of a precise desert classification and opacity regarding associated terms might confound policy and management goals in deserts, especially due to the considerable human land-use, climate change, and land-degradation challenges facing the world’s deserts. Formalizing a new classification of deserts might help to advance accurate research, conservation and policy goals in deserts. Given the cross-sector implications, such an effort might be appropriately led by an international body such as UNEP or coordinated through platforms like IPBES or UNCCD, with input from both ecological and policy communities. A standardized framework would not only clarify communication but also improve the targeting of interventions in desert regions.

In addition to climatic and biophysical variables, advancing a more effective desert classification also requires recognizing deserts as social–ecological systems (SES)—places where human and environmental dynamics are deeply intertwined. Feedbacks between ecological processes (such as vegetation cover or water availability) and socioeconomic forces (such as land tenure, infrastructure development, or resource access) vary across deserts, influencing resilience and degradation risks in context-specific ways (Reynolds et al. 2007; Ostrom 2009; IPBES 2018). Incorporating SES perspectives alongside ecological criteria offers a more holistic basis for research, conservation, and land management in deserts—particularly under intensifying pressures from climate change, global markets, and shifting land-use patterns (Turner et al. 2003; Cherlet et al. 2018; Lewin et al. 2021).

### Confusion surrounding ‘desertification’

**Fig. 3:**
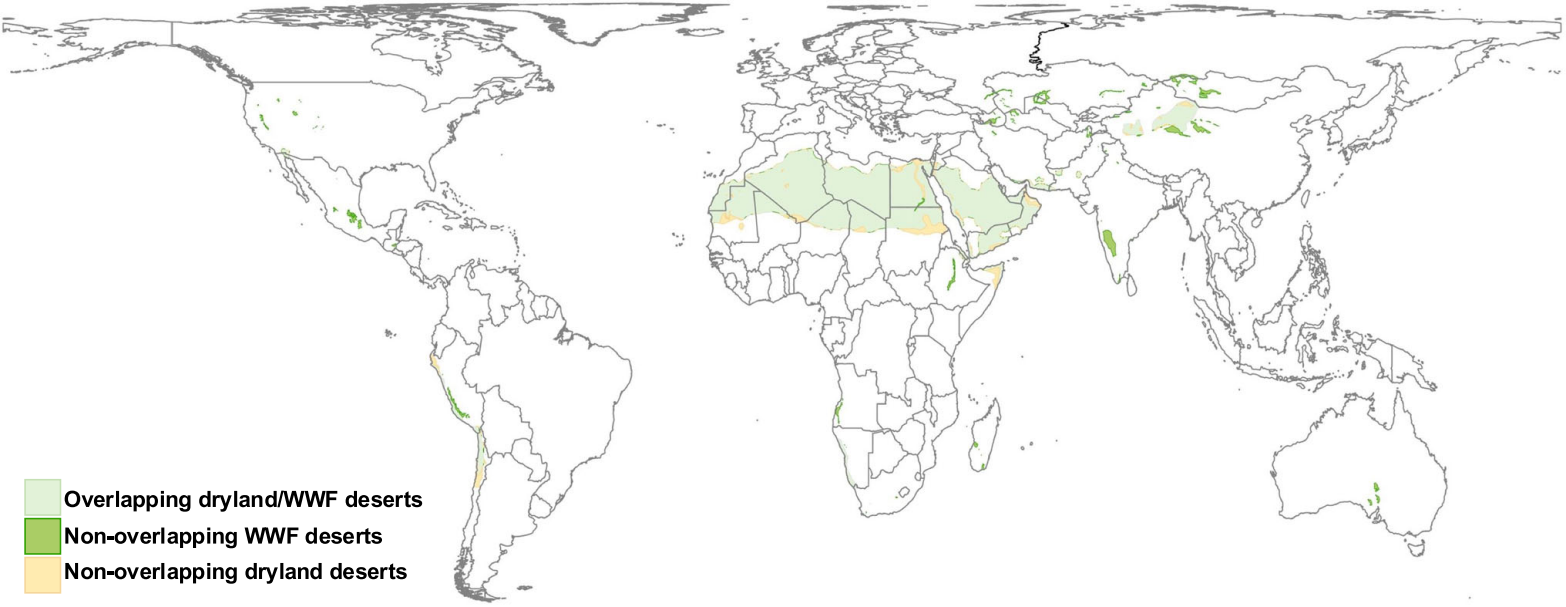
Hyperarid dryland deserts and WWF deserts excluded (~ 9.8 million km^2^) from the UNCCD definition of desertification, which applies only to the degradation of arid, semiarid, and dry subhumid drylands

While deserts are often misunderstood and misclassified, the term ‘desertification’ further complicates effective land management by conflating the natural characteristics of deserts with human-induced degradation. The United Nations Convention to Combat Desertification (UNCCD) and Intergovernmental Panel on Climate Change (IPCC) define desertification as the degradation of biologic and economic productivity in arid, semiarid, and dry subhumid drylands resulting from factors including climatic variation and human activities (IPCC 2019; Vicente-Serrano et al. 2024). However, this definition excludes approximately 9.8 million km^2^ of hyperarid drylands and WWF-defined deserts (Fig. 3), reinforcing blind spots in land-use policy and overlooking ongoing degradation in desert regions. These neglected regions represent vast, productive, and diverse deserts (Martínez-Valderrama et al. 2020), which are particularly sensitive and vulnerable to human overuse coupled with rapidly growing populations (Reynolds et al. 2007; IPBES 2018). Importantly, significant land degradation can occur even in hyperarid deserts, particularly in areas of concentrated human activity and resource extraction. For example, in the Arava Valley of Israel, where intensive agriculture in a hyperarid setting has led to substantial pressures on soil and water resources (Lewin et al. 2021). Therefore, it is crucial that deserts are not overlooked when considering global evaluations of land degradation, yet currently there is no global or institutional body focused exclusively on deserts for policy and management goals (UNEP 2006). Furthermore, increases in aridity are primarily projected around desert fringes and non-desert drylands, while few studies incorporate existing hyperarid deserts in assessments of land degradation or environmental change (Huang et al. 2017; Song et al. 2018; Berdugo et al. 2020; Burrell et al. 2020). Yet, projections suggest that hyperarid zones could cover nearly 12.6% of the earth’s terrestrial surface by 2100 (Huang et al. 2016)—underscoring the growing importance of understanding these understudied regions. Desertification infers that desertified land should resemble a natural desert or that deserts expand naturally (Manguet 1991). The notion of deserts expanding of their own accord (for example, by high winds and sands) is no longer widely regarded when accounting for natural variation and seasonal cycles—common ecosystem functions in desert systems (Tucker and Dregne 1991; Dronin 2023). While moderate climatic dryland expansion is projected due to hotter and drier conditions (Huang et al. 2016; Burrell et al. 2020), with potential shifts to dryland ecosystem mechanisms in some regions—it is crucial not to confound increased warming with the fallacy of “the encroaching desert” or to wrongly refer to this phenomenon as desertification. This confusion stems largely from interpretation and popular usage, rather than flaws in the scientific or policy definitions themselves; as the UNCCD defines desertification specifically as land degradation in arid, semiarid, and dry subhumid areas due to climatic and anthropogenic pressures—not the physical spread of deserts. Unfortunately, these terms are frequently used interchangeably in the literature, policy discussions, and media, which can cause confusion about the underlying processes and their implications (Thomas and Middleton 1994; Reynolds and Smith 2002; Verón et al. 2006; UNCCD 2020). As described above, deserts are characterized by a complex set of geographical, biological and evolutionary attributes rather than being merely characterized by simple climatic measures such as temperature (Spinoni et al. 2021). The term desertification, as colloquially used, thus diminishes the intrinsic value of natural deserts—treating the term ‘desert’ as a pejorative—and obscures the ability to effectively identify and address drivers of degradation in actual desert regions (Vogt et al. 2011; Davis 2016). Moreover, most studies referencing desertification extend its use beyond deserts (Fig. 3) to a range of ecoregions and in some cases apply the term more broadly to contexts unrelated to drylands altogether, such as tropical/subtropical, forest and aquatic ecosystems (Safriel 2009) or “food deserts” (areas with limited access to affordable and nutritious food; Cattivelli 2022)—highlighting the varied and sometimes inconsistent use of the term in the literature (Reynolds 2021). Consequently, using the term desertification in efforts to evaluate and mitigate land degradation may risk overlooking valuable ecological and human systems in deserts—particularly in regions that fall outside the UNCCD scope. Moreover, the term itself can reinforce negative stereotypes of deserts as degraded or unproductive, rather than recognizing them as unique, biodiverse, and inhabited landscapes. Rather than abandoning the term altogether, we suggest the use of complementary language that explicitly includes land degradation in hyperarid deserts. This approach preserves the utility of ‘desertification’ as an internationally recognized concept, while also expanding the conversation to address currently overlooked ecosystems and support more inclusive and effective land management strategies (Safriel 2007; D’ordorico 2013; Casek et al. 2015; Cherlet et al. 2018).

## Conclusion

Misunderstandings surrounding the global delineation of deserts and the concept of desertification may hinder scientific, policy, and development initiatives, and negatively influence social perspectives regarding desert systems. We aim to clarify such misconceptions by highlighting that strictly climatic or ecological approaches to classifying deserts fail to capture the diversity and complexity of recognized desert regions. A new comprehensive desert classification is needed, encompassing a range of climatic, biophysical, and ecological variables to better guide development, conservation, and policy goals in deserts. We also show that desertification is a misapplied term suggesting the erroneous expansion of deserts, while overlooking land degradation in vast desert regions. The misclassification of deserts coupled with the misrepresentation of desertification ultimately obscures the ability to effectively address the drivers of degradation in desert regions inclusively. Recognizing deserts as social–ecological systems—shaped by feedbacks between environmental processes and human activities—can further support the design of land-use strategies that are responsive to context-specific vulnerabilities and resilience. We hope to challenge negative perceptions toward desert systems, shifting both policy and scientific discussions to recognize deserts as diverse, ecologically rich, and culturally significant regions that contribute meaningfully to global sustainability and biodiversity.

## Materials and methods

### Comparing desert classifications

We evaluated the spatial distribution and overlap of two existing desert delineations based on climatic versus ecological approaches to classifying deserts. (1) Dryland deserts as classified by the United Nations Environment World Conservation Monitoring Centre (UNEP-WCMC 2007)—are based on aridity values defined as the ratio of precipitation to potential evapotranspiration (P/PET). Deserts are defined as hyperarid (< 0.06 P/PET) and arid (0.06–0.2 P/PET) dryland subtypes (herein ‘dryland deserts’) (UNEP 2006). Data were obtained using the ‘Drylands Dataset’.^1^ (2) The World Wildlife Fund for Nature (WWF) classifies the ‘Deserts and Xeric Shrublands’ biome as a distinct desert assemblage of natural biotic communities with shared environmental conditions (herein ‘WWF deserts’) (Dinerstein et al. 2017). Data were obtained using the Resolve10 dataset from.^2^ All data were processed using ArcGIS (v10.8.1.) and projected with an equal-area Behrmann projection. We overlaid dryland deserts with WWF deserts to determine the relative area of overlapping and non-overlapping desert regions. We overlaid arid, semiarid, and dry subhumid drylands with hyperarid drylands and WWF deserts to determine the extent of hyperarid drylands and WWF-defined deserts falling outside the UNCCD definition of ‘desertification,’ which applies only to degradation in arid, semiarid, and dry subhumid zones (Fig. 3).

### Policy implications for human land-use and biodiversity conservation in deserts

To evaluate human population numbers in deserts, we overlaid 2020 human population counts data from the Gridded Population of the World collection (GPWv4; CIESIN 2018) with overlapping and non-overlapping dryland and WWF deserts (Table 1). Human population clusters were categorized using administration units (Fig. 1). All data obtained from the Socioeconomic Data and Application Center (SEDAC).^3^ Due to variability among the relative size and spatial proximity of administration units and reporting differences (based on over 13.5 million global input administrative units), we combined administration units by geographic region. For example, in North America, at the second administrative level (for example, subunits within Maricopa County, Arizona, are aggregated and their populations summed). In Asia, Africa, Europe, and South America, we combined data at the third administration level where data were available (for example, San Juan de Lurigancho in the city of Lima). However, many countries reported coarser first or second administrative units only (for example, Anseba Region in Eritrea). We show representative centers of geographic coordinates between synonymous administration units, showing large population clusters only in Fig. 1 (> 500 000 people). We labeled clusters of several adjacent administrative units under recognizable city names (for example, Cairo). All data are provided in Supplementary Data 1.

To evaluate land-use patterns, we used data from Sentinel-2 10m land-use/land-cover time series for the year 2023 derived from ESA (Karra et al. 2021). We focus on ‘Crops’ and ‘Built Area’ class definitions. Crops (Value 5) are categorized as human planted/plotted cereals, grasses, and crops not at tree height (for example, corn, wheat, soy, fallow plots of structured land). Built area (Value 7) is categorized as human-made structures, major road and rail networks, large homogenous impervious surfaces including parking structures, office buildings and residential housing (for example, houses, dense villages/towns/cities, paved roads, asphalt). Data from.^4^

To determine the species richness of terrestrial vertebrate species in deserts, we obtained extent of occurrence polygons for all breeding, extant and native amphibians and mammals from the IUCN (v.6.3),^5^ birds from BirdLife International (v.4),^6^ and reptiles from the Global Assessment of Reptile Distributions (GARD v.1.7; Roll et al. 2017; de Oliveira et al. 2022). We defined desert species as those with ≥ 50% of their global ranges occurring in non-overlapping dryland or WWF deserts (by overlaying species polygons with respective desert regions; Lewin et al. 2024). Endemic species are defined as those with > 99% of their global distributions occurring in non-overlapping dryland or WWF deserts (Fig. 2 and Supplementary Data 2). We evaluated the threat status of desert species in non-overlapping dryland and WWF deserts by grouping species identified as threatened using the IUCN Red List categories—vulnerable (VU), endangered (EN), and critically endangered (CR), and by grouping species in non-threatened categories—near threatened (NT), least concern (LC). All data obtained from footnote 5.

## Supplementary Information

Below is the link to the electronic supplementary material.

Supplementary file1 (DOCX 502 KB)

Supplementary file2 (DOCX 60 KB)

## Footnotes

1 https://resources.unep-wcmc.org/products/789fcac8959943ab9ed7a225e5316f08

2 https://ecoregions.appspot.com/.

3 https://www.arcgis.com/home/item.html?id=a9fea1ecd1ba4f7db80a0f667fbc508b.

4 https://livingatlas.arcgis.com/landcoverexplorer/#mapCenter=52.13765%2C54.76633%2C2.923296114067695&mode=step&timeExtent=2017%2C2023&year=2023&landCover=Crops.

5 www.iucnredlist.org/.

6 http://datazone.birdlife.org/.
